# Immigration counter‐acts local micro‐evolution of a major fitness component: Migration‐selection balance in free‐living song sparrows

**DOI:** 10.1002/evl3.214

**Published:** 2021-01-15

**Authors:** Jane M. Reid, Peter Arcese, Pirmin Nietlisbach, Matthew E. Wolak, Stefanie Muff, Lisa Dickel, Lukas F. Keller

**Affiliations:** ^1^ Centre for Biodiversity Dynamics NTNU Trondheim Norway; ^2^ School of Biological Sciences University of Aberdeen Aberdeen UK; ^3^ Forest & Conservation Sciences University of British Columbia Vancouver British Columbia Canada; ^4^ School of Biological Sciences Illinois State University Normal Illinois USA; ^5^ Department of Biological Sciences Auburn University Auburn Alaska USA; ^6^ Department of Mathematical Sciences NTNU Trondheim Norway; ^7^ Department of Evolutionary Biology & Environmental Studies University of Zurich Zurich Switzerland; ^8^ Zoological Museum University of Zurich Zurich Switzerland

**Keywords:** Additive genetic variance, dispersal, evolutionary rescue, fitness, gene flow, genetic groups, immigration load, migration‐selection balance, quantitative genetics

## Abstract

Ongoing adaptive evolution, and resulting “evolutionary rescue” of declining populations, requires additive genetic variation in fitness. Such variation can be increased by gene flow resulting from immigration, potentially facilitating evolution. But, gene flow could in fact constrain rather than facilitate local adaptive evolution if immigrants have low additive genetic values for local fitness. Local migration‐selection balance and micro‐evolutionary stasis could then result. However, key quantitative genetic effects of natural immigration, comprising the degrees to which gene flow increases the total local additive genetic variance yet counteracts local adaptive evolutionary change, have not been explicitly quantified in wild populations. Key implications of gene flow for population and evolutionary dynamics consequently remain unclear. Our quantitative genetic analyses of long‐term data from free‐living song sparrows (*Melospiza melodia*) show that mean breeding value for local juvenile survival to adulthood, a major component of fitness, increased across cohorts more than expected solely due to drift. Such micro‐evolutionary change should be expected given nonzero additive genetic variance and consistent directional selection. However, this evolutionary increase was counteracted by negative additive genetic effects of recent immigrants, which increased total additive genetic variance but prevented a net directional evolutionary increase in total additive genetic value. These analyses imply an approximate quantitative genetic migration‐selection balance in a major fitness component, and hence demonstrate a key mechanism by which substantial additive genetic variation can be maintained yet decoupled from local adaptive evolutionary change.

**Impact Summary**

How fast can evolution occur in wild populations? What processes can facilitate rapid evolution, or else prevent evolution from occurring? Answering these questions is central to understanding how species might adapt to current rapid environmental changes.

Evolutionary change in any population results from a combination of genetic variation and natural selection, following Darwin's basic principle. Yet, the pace of local evolution could be dramatically altered by the arrival of immigrants from other populations, which could be better or less well adapted to local conditions. Immigrants might facilitate local adaptive evolution by importing beneficial genetic variants, or might impede or even reverse local adaptive evolution by importing detrimental genetic variants that negate local evolutionary advances. We need to distinguish between these possibilities in order to predict how evolution will proceed; yet we still know surprisingly little about the impacts of natural immigration on the critical genetic variation that underlies adaptive evolution in nature.

We applied advanced “quantitative genetic” analyses to >25 years of pedigree (i.e., family‐tree) data from a Canadian population of song sparrows to quantify the effects of recent immigration on local adaptive evolution. We demonstrate that, during the past quarter century, there has been a substantial local evolutionary increase in the probability that a juvenile sparrow will survive to adulthood. However, we show that this recent evolutionary advance has been wiped out by detrimental genetic effects imported by recent immigrants, that reduce local survival. Song sparrows are therefore caught in a balance between the conflicting evolutionary forces of local natural selection and gene flow resulting from immigration.

These results illustrate an important route by which rapid adaptive evolution can be constrained, despite the presence of substantial genetic variation and strong natural selection. Predictions of the rate of contemporary evolution in nature must consequently consider the complex genetic consequences of immigration.

It is increasingly clear that major fitness components, and measures of fitness, often exhibit moderate additive genetic variance (V_A_) in wild populations (Brommer et al. 2007; Postma 2014; Hendry et al. 2018; Sheth et al. 2018; Wolak et al. 2018). This negates any simple expectation that V_A_ will be rapidly eroded by selection and hence be very small (Kirkpatrick 2009; Shaw and Shaw 2014; Shaw 2019). Such V_A_ in fitness provides the critical raw material for ongoing evolution, and for rapid “evolutionary rescue” of declining populations (Carlson *et al*. 2014; Shaw and Shaw 2014; Shaw 2019). But, by corollary, such V_A_ can also open a puzzle that requires attention. Namely, if there is V_A_ in fitness, or in major underlying fitness components, then mean additive genetic values should increase across consecutive generations under directional selection. Any resulting micro‐evolutionary increases in absolute fitness should cause increasing population growth (once the finite rate of increase λ exceeds one, Kirkpatrick 2009; Bell 2017). Yet, phenotypic increases in fitness and population growth cannot occur indefinitely, and short‐term increases are not generally observed in wild populations where V_A_ in fitness is estimated to be present (Kirkpatrick 2009; Shaw 2019).

This situation mirrors a common wider discrepancy, where predicted micro‐evolutionary responses in mean phenotypic trait values in wild populations are not observed (Merilä *et al*. 2001; Bonnet et al. 2017; Pujol et al. 2018; Shaw 2019). When the focal trait is fitness, or a major fitness component that is consistently positively correlated with fitness and hence under consistent directional selection, this implies that available V_A_ has been over‐estimated and/or that there is some form of counter‐gradient that decreases mean phenotypic value sufficiently to obscure an underlying micro‐evolutionary increase (e.g. Frank and Slatkin 1992; Merilä *et al*. 2001; Pujol et al. 2018). Negative environmental counter‐gradients are often invoked, and may indeed exist due to detrimental climate and/or habitat change or, in the context of fitness, increasing population density (Frank and Slatkin 1992; Merilä *et al*. 2001; Gomulkiewicz and Shaw 2013; Shaw 2019).

However, additional constraints on, or drivers of, micro‐evolutionary responses to selection can arise in spatially‐structured populations. Here, local V_A_ in fitness or any fitness component in any sub‐population could be increased by gene flow stemming from immigration (i.e., incoming dispersers, Phillips 1996; Gomulkiewicz and Shaw 2013; Carlson *et al*. 2014; McDonald and Yeaman 2018). Yet, such gene flow could reduce local mean fitness rather than straightforwardly facilitate local adaptive micro‐evolution (Bolnick and Nosil 2007; Kremer et al. 2012). This situation arises if immigrants have low additive genetic values for local fitness. Continuing introgression could then counteract local micro‐evolutionary increases that would otherwise be expected. Gene flow could thereby generate an additive genetic counter‐gradient that maintains phenotypic stasis despite nonzero V_A_ and consistent directional selection, yielding a quantitative genetic “migration‐selection balance” (e.g., Phillips 1996; Lenormand 2002). Local distributions of additive genetic values could then be somewhat skewed toward the immigrants’ mean, potentially further altering evolutionary outcomes (Yeaman and Guillaume 2009; Huisman and Tufto 2012; Débarre et al. 2015).

There are multiple nonexclusive reasons why immigrants might have low additive genetic values for local fitness, thereby counteracting local micro‐evolution. If effective gene flow within heterogeneous landscapes is relatively infrequent there could be a local adaptation, meaning that immigrants’ alleles are unsuited to conditions in recipient sub‐populations (Phillips 1996; Lenormand 2002; Bolnick and Nosil 2007; Hereford 2009; Savolainen et al. 2013; McDonald and Yeaman 2018). Immigrants could comprise non‐random subsets of their natal sub‐populations, for example, if genetically uncompetitive individuals are forced to emigrate (e.g., Edelaar and Bolnick 2012). Indeed, if there is V_A_ in propensity for dispersal, immigrants’ descendants would be more likely to emigrate in turn, and consequently have low additive genetic values for local fitness measured as their contribution to the original immigrant's recipient sub‐population (Doligez and Pärt 2008). Given these broad possibilities, the degree to which naturally‐occurring immigration does in fact increase local additive genetic variance and skew yet counteract local micro‐evolutionary increases in fitness, and thereby constrain rather than enhance the potential for “evolutionary rescue,” needs to be explicitly quantified in wild populations (Bolnick and Nosil 2007; Yeaman and Guillaume 2009; Edelaar and Bolnick 2012; Kremer et al. 2012; Gomulkiewicz and Shaw 2013; Carlson *et al*. 2014; Bell 2017).

From a quantitative genetic perspective, migration‐selection balance can be conceptualized as approximately constant mean additive genetic values in the face of local selection and movement, whereas underlying allele frequencies may still be dynamic (e.g., Phillips 1996; Yeaman and Guillaume 2009; Huisman and Tufto 2012; Débarre et al. 2015). This perspective may typically be appropriate for major fitness components, which are likely to be highly polygenic with scope for genetic redundancy (Phillips 1996; Huisman and Tufto 2012), although some large effect loci might also be expected (Yeaman and Guillaume 2009). Any individual i's total additive genetic value (*u*_i_) can then be specified as a function of introgression between defined groups of recent immigrants versus preexisting local natives (termed “genetic groups”). Specifically, *u*_i_ can be partitioned into a breeding value (*a*_i_, i.e., individual i's expected deviation from the group mean due to additive genetic effects), plus the difference in mean value between the defined immigrant and native genetic groups (the genetic group effect g) multiplied by the expected contribution of the immigrant genetic group to individual i's genome (its immigrant genetic group coefficient *q*_i_, Equation 1, Quaas 1988; Westell et al. 1988; Wolak and Reid 2017):

(1) $$u_{i}=a_{i}+g.q_{i}$$

Equation 1 implies that immigration will increase the variance in total additive genetic value (V_U_, i.e., the population variance in *u*_i_) above V_A_ (i.e., the population variance in *a*_i_) as long as g≠0 and there is some among‐individual variance in *q*_i_ (Reid and Arcese 2020; Supporting Information S1). While breeding values *a*_i_ are typically assumed to be normally distributed, Equation 1 also implies that the distribution of total additive genetic values *u*_i_ could be skewed, to a degree that also depends on g and the variance and skew of *q*_i_ (Supporting Information S1). Further, Equation 1 indicates how immigration could counter‐act any expected micro‐evolutionary increase in mean breeding value (i.e., mean *a*_i_) between generations due to local selection. For example, if g<0, meaning that immigrants have a lower mean value than preexisting natives, and the mean genetic contribution of immigrants (i.e., mean *q*_i_) increased between generations (e.g., due to further immigration) then the product g.*q*_i_ would become more negative. There might then be little or no change in mean *u*_i_ despite the underlying micro‐evolutionary increase in mean *a*_i_.

This formulation (Equation 1) has the major advantage that the key parameters can be explicitly estimated in wild populations (using “genetic groups animal models,” Wolak and Reid 2017; Wolak et al. 2018; Muff et al. 2019; Reid and Arcese 2020). Such models can extend standard pedigree‐based quantitative genetic analyses by defining distinct groups of recent immigrants and native founders, and thereby explicitly estimate the additive genetic effects of immigrants. Initial models fitted to long‐term data from song sparrows (*Melospiza melodia*) revealed a negative additive genetic effect of recent immigrants (i.e., g<0) on local juvenile survival to adulthood, and consequently on overall individual fitness measured as total local lifetime reproduction from hatch (Wolak et al. 2018). However, the resulting predictions that immigration and resulting gene flow increase total local additive genetic variation and skew, but simultaneously counteract micro‐evolutionary increases in mean breeding value to yield some degree of quantitative genetic migration‐selection balance, have not been explicitly tested in any wild system.

Accordingly, we used the song sparrow data to quantify four key effects that determine how local micro‐evolution and immigration combine to shape local evolutionary outcomes. First, we tested whether mean breeding value (i.e., mean *a*_i_) for local juvenile survival increased over an observed 26‐year timeframe, representing micro‐evolutionary change that should be expected given non‐zero V_A_ in a major fitness component. Second, we tested whether the overall additive genetic effect of recent immigrants (i.e., mean g.*q*_i_) became increasingly negative over the same timeframe, generating an additive genetic counter‐gradient. Third, we tested whether mean total additive genetic value (i.e., mean *u*_i_) changed across years, or whether the negative effect of immigration counteracted local micro‐evolution in mean *a*_i_ to generate approximate migration‐selection balance. Finally, we quantified the degree to which the variance and skew in total additive genetic value *u*_i_ exceeded those in *a*_i_, and hence to which natural immigration reshaped the total local additive genetic variation that is potentially available to generate further micro‐evolutionary responses to selection.

## Methods

### FIELD SYSTEM

Pedigree and life‐history data have been collected in a song sparrow population inhabiting Mandarte island BC, Canada (ca. 6 hectares) since 1975, allowing advanced quantitative genetic analyses to test key evolutionary hypotheses (Reid et al. 2011; Germain et al. 2016; Wolak et al. 2018; Reid and Arcese 2020). Briefly, song sparrows are primarily socially monogamous open‐nesting passerines. Pairs typically rear up to 3 sequential broods of 1–4 chicks during April‐August each year. Each year, all territories and nests were monitored, and all chicks surviving to or beyond 6 days post‐hatch were marked with unique combinations of colored plastic and metal rings to allow individual identification. All territories were then systematically surveyed to identify chicks that survived to independence from parental care (approximately 24 days post‐hatch, Smith et al. 2006). Comprehensive island‐wide surveys were undertaken in April each year to identify all individuals hatched the previous year that had locally survived to adulthood (i.e., age approximately 1 year, Smith et al. 2006), with annual resighting probability close to one. All field protocols were approved by the University of British Columbia Animal Care Committee.

**Figure 1:**
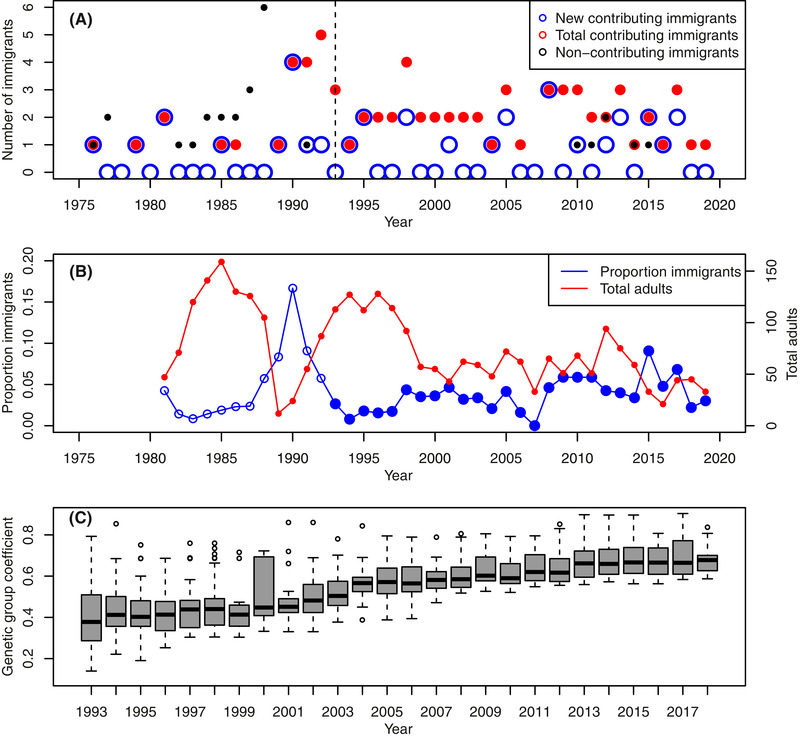
Summaries of numbers of immigrant song sparrows and their expected genetic contributions. **(A)** Numbers of immigrants observed in each year that contributed to current analyses (i.e., ≥1 descendant hatched during 1993–2018, period right of the dashed line). Blue circles denote the total newly arrived immigrants in each year. Red points denote the total contributing immigrants alive in each year, including surviving immigrants that had arrived previously. Black points denote immigrants alive in each year that did not contribute to current analyses (i.e. no descendants hatched during 1993–2018). These comprise immigrants that arrived before 1993 whose lineages did not persist, and more recent immigrants that did not breed. **(B)** Total number of adults alive in April each year (red, secondary *y*‐axis), and the proportion of these adults that were immigrants (blue, primary *y*‐axis). Data are shown from 1981 because the total number of immigrants prior to that is unknown (because any surviving immigrants that arrived before 1975 are not identifiable). Filled blue symbols highlight focal cohorts spanning 1993–2018. **(C)** Distributions of individual genetic group coefficient (*q*_i_) across individuals hatched in each year during 1993–2018. Boxplots show the median (central thick line), first and third quantiles (box limits), 1.5× interquartile range (whiskers), and outliers (points). Zero individuals had two immigrant parents (and hence *q*_i_ = 1). Full summary statistics are in Supporting Information S3. Note that x‐axis scales differ between panels (A) and (B) versus (C).

Mandarte's song sparrows are typically sedentary, and adults are highly philopatric to their breeding territories across years. However, since Mandarte lies within a large natural meta‐population, there are incoming immigrants (mean approximately 1 year^−1^, range 0–4, Fig. 1A). These immigrants, initially identifiable as unringed adults given that all Mandarte‐hatched individuals are color‐ringed, were mist‐netted and color‐ringed soon after arriving. There is presumably some unobserved juvenile emigration, which cannot be distinguished from mortality. However, local juvenile survival probabilities from independence to age one year (i.e., recruitment) are relatively high (overall means: 0.23 and 0.31 for females and males during 1993–2018) given that true first‐winter mortality will be high. This implies that juvenile emigration probabilities must typically be relatively low (Supporting Information S2). Indeed, surveys of surrounding islands during 1998–2004 (Wilson and Arcese 2008) revealed no Mandarte‐ringed emigrants, indicating that any surviving emigrants typically leave the local system. Such permanent emigration has the same immediate effect on local distributions of genetic values as mortality (e.g., Bonnet et al. 2017).

### QUANTITATIVE GENETIC MODEL STRUCTURE

We fitted a univariate genetic groups animal model to estimate V_A_ in local juvenile survival from independence to adulthood (an individual binary trait), and estimate *a*_i_ for all individuals, by fitting random individual effects with variance‐covariance structure defined by pedigree‐derived relatedness. The model also included random natal brood and year effects to estimate variances associated with common environmental effects within broods and years (full model details, Supporting Information S4).

The model included a fixed regression on individual immigrant genetic group coefficient *q*_i_, with *q*_i_ values calculated from pedigree data. The estimated regression slope is the genetic group effect g, which equals the difference in mean additive genetic value between defined immigrant and native genetic groups (with the native mean taken as zero by convention, Wolak and Reid 2017; Wolak et al. 2018). The same model also included four further fixed effects (full details, Supporting Information S3). First, we modeled a regression on the individual coefficient of inbreeding (*f*_i_) to account for known inbreeding depression (Reid et al. 2014; Wolak et al. 2018), since unmodeled inbreeding depression can bias estimates of V_A_ (Reid and Keller 2010). Second, since song sparrows can rear multiple sequential broods within each summer, offspring hatch dates can vary by up to 3 months. We therefore modeled a regression on clutch date (i.e., date on which the first egg in each individual's natal clutch was estimated to have been laid) to account for any variation in individual local juvenile survival due to parental breeding date (i.e., a parental rather than individual effect). Third, we modeled a regression on year (as a continuous variable) to account for any linear environmentally‐induced phenotypic change in juvenile survival across years that could potentially bias estimates of genetic change (Postma and Charmantier 2007). Fourth, we modeled a fixed effect of sex to capture a previously observed difference in mean local survival between females and males (Reid 2012; Wolak et al. 2018). Since these previous analyses showed a strong positive cross‐sex genetic correlation, for current purposes we treated juvenile survival as a single quantitative trait encompassing both sexes.

### PEDIGREE, GENETIC GROUPS, AND PHENOTYPIC DATA

Genetic groups animal models designed to quantify additive genetic consequences of immigration require assignment of pedigree founders (i.e., individuals with unknown parents) to “immigrant” and “native” groups, and estimate effects for the respective defined base populations (Wolak and Reid 2017). To generate the required pedigree, the social parents of all offspring hatched since 1975 (i.e., the female and male providing parental care) were identified through comprehensive territory surveys. From 1993, all offspring and potential parents were genotyped at 13 highly polymorphic microsatellite markers, allowing individuals’ true genetic parents to be identified with very high individual‐level confidence (typically >0.99, Sardell et al. 2010). Paternities of offspring hatched during 1993–2013 were further verified using 160 microsatellite markers (Nietlisbach et al. 2015). Hence, the full available pedigree spanning 1975–2018 comprises complete accurate genetic parentage data for 1993–2018, correcting for ∼28% extra‐pair paternity, and primarily social parentage data for 1975–1992 (e.g., Sardell et al. 2010; Reid et al. 2011; Reid et al. 2014; Nietlisbach et al. 2015, 2017; Wolak et al. 2018). This pedigree, which contains numerous known half‐ and full‐siblings reared in the same and different brood environments, with substantial immigration‐induced variance in kinship that is uncorrelated with fine spatial location, provides substantial power for quantitative genetic analyses (Supporting Information S3; Germain et al. 2016; Wolak et al. 2018; Reid and Arcese 2020).

To minimize any bias due to remaining paternity error in the pre‐1993 pedigree, phenotypic data (i.e., local juvenile survival) were restricted to 26 cohorts hatched on Mandarte during 1993–2018. This comprises all individuals whose parents were genetically verified, with local survival to adulthood observed by April 2019. Since mean generation time is approximately 2.5 years (Reid et al. 2019), this represents approximately ten generations on average. The full pedigree was pruned to the focal phenotyped individuals and all assigned ancestors back to 1975, thereby utilizing all available pedigree information (Supporting Information S3). The immigrant genetic group was defined as all individuals that arrived on Mandarte since 1975 and contributed some descendants to the phenotyped individuals hatched since 1993 (and hence were retained in the pruned pedigree, Supporting Information S4). The native genetic group was defined as all other retained individuals with unknown parents, primarily comprising individuals present in 1975 that contributed phenotyped descendants (Wolak et al. 2018). This structure ensured that mean *q*_i_ and *f*_i_ in the first focal cohort in 1993 were non‐zero, thereby minimizing spurious collinearities in *q*_i_, *f*_i_ and kinship and resulting bias that would arise given insufficient initial pedigree depth (Supporting Information S3). This allows biologically meaningful evaluation of the degree to which genetic effects of immigration counteracted local micro‐evolution during the focal period. Mean *q*_i_ could potentially have increased or decreased during 1993–2018, depending on patterns of effective gene flow and local fitness of immigrants’ descendants. Analyses of genotypic data for 160 microsatellites for 17 immigrants that arrived during 1993–2013 strongly supported the underlying assumption that immigrants were typically unrelated to the existing Mandarte population at the time of arrival (relative to the local pedigree base population). One individual that was apparently distantly related contributed very little to variation in *q*_i_, meaning that alternative assumptions regarding its ancestry and hence genetic group membership did not perceptibly alter the estimate of g.

### ANALYSES

Individuals’ expected values of *q*_i_ and *f*_i_, and the inverse relatedness matrix, were calculated from the pruned pedigree using standard algorithms implemented in package nadiv (Wolak 2012) in R version 3.5.1 (R Core Team 2018). The genetic groups animal model (with binomial errors and logit link) was fitted in a Bayesian framework using package MCMCglmm (Hadfield 2010), facilitating latent‐scale inference for the non‐Gaussian phenotype and estimation of full posterior distributions of key derived parameters integrating over uncertainty. Priors on fixed effects were normally distributed with mean zero and large variance (10^10^), and hence largely uninformative. To facilitate mixing, priors on variance components were parameter‐expanded, giving proper Cauchy priors for the standard deviations. Posterior means, modes, and 95% highest posterior density credible intervals (CIs) for variance components and fixed effects were summarized across 3000 posterior samples with autocorrelation <0.05 (model code, Supporting Information S4).

The genetic groups animal model directly returns posterior distributions of V_A_ and g. Posterior distributions of *a*_i_ for all individuals were also extracted. Full posterior distributions of individual *u*_i_ were then computed following Equation 1, since each individual's expected value of *q*_i_ is known given the observed pedigree (Wolak and Reid 2017). Full posterior distributions of key derived parameters were then computed and summarised. These primarily comprise the slopes of regressions of breeding value (*a*_i_), immigrant genetic effect (g.*q*_i_), and total additive genetic value (*u*_i_) on year across the focal cohorts (i.e., hatched 1993–2018). Full posterior distributions of the cohort population variance and skew in *a*_i_ and *u*_i_, the differences between the variance and skew in *u*_i_ versus *a*_i_, and the regressions of these quantities on year, were also computed and summarized. These statistics should be unbiased given that selection is ignorable (sensu Sorensen et al. 2001), since all individuals in each cohort were retained in the dataset. In contrast, means, variances, and skew of posterior modes or means of individual *a*_i_ and *u*_i_ are likely to be biased (e.g. Hadfield et al. 2010) and were not used for inference. Detailed checks revealed no evidence of major multi‐collinearities, sampling correlations, or other structures in the data that could substantially confound or bias key results (Supporting Information S5).

An increase in mean *a*_i_ across years would provide evidence of evolutionary change, but would not directly demonstrate a directional micro‐evolutionary response to selection as opposed to drift (Hadfield et al. 2010). To test whether observed temporal change in mean *a*_i_ exceeded that expected solely due to drift (given the observed pedigree), we simulated values of *a*_i_ on the pedigree with founder values (for both natives and immigrants) drawn from a normal distribution with mean zero and variance equal to each posterior sample of V_A_ (full details, Supporting Information S4). We computed the posterior distributions of the slope of the regression of randomised *a*_i_ on year, and of the difference in regression slope given estimated versus randomised *a*_i_ (across paired estimates given each posterior sample of V_A_). The proportion of positive differences gives the posterior probability that an observed increase in *a*_i_ exceeds that which could arise by chance given the observed pedigree (Supporting Information S4, Hadfield et al. 2010).

### PHENOTYPIC SELECTION AND EFFECT SIZES

Since individuals that do not locally survive to adulthood by definition leave zero local offspring, while most individuals that survive to adulthood do leave some offspring, local phenotypic selection gradients on local juvenile survival must be consistently positive. Nevertheless, to illustrate the magnitude of selection, we calculated selection gradients as the slopes of regressions of individual reproductive success on juvenile survival within each cohort (full details, Supporting Information S6). However, we did not currently attempt to combine estimates of V_A_ and selection to quantitatively predict micro‐evolutionary change in juvenile survival; such projections are particularly challenging given dynamic inbreeding, immigration and resulting potential for changing and skewed distributions of additive genetic values (see Discussion).

To indicate biological effects of estimated changes in *a*_i_ and g.*q*_i_ between 1993 and 2018 on juvenile survival probability, and hence evaluate effects of local micro‐evolutionary responses and immigration, posterior distributions of latent (logit) scale estimates for these years were back‐transformed onto the phenotypic scale taking mean values of all other modeled fixed effect variables. As an additional summary statistic, the posterior distribution of the latent‐scale heritability of juvenile survival was calculated as the ratio of V_A_ to the sum of all estimated variance components (Wolak et al. 2018). The numbers of immigrants present in the population in each year, and the proportion of adults that were immigrants, were summarized.

## Results

### DATA SUMMARY

In total, 2478 song sparrows hatched on Mandarte during 1993–2018 and had observed phenotypes for local survival to adulthood (mean 95.3 ± 35.9 SD individuals per year, range 22−182, representing 1109 broods with means of 42.6 ± 15.6 SD broods per year and 2.2 ± 0.9 SD individuals per brood). The grand mean standardized phenotypic selection gradient on juvenile survival across cohorts was 1.8 (range 0.8−4.1, Supporting Information S6).

The pruned pedigree totaled 2722 informative individuals (2478 phenotyped individuals and 244 additional ancestors). There were 15 defined native founders, and 33 informative immigrants (25 females, 8 males) that arrived during the full study period (Fig. 1A). The total number of immigrants present, and the proportion of adults that were immigrants, was temporarily high following a local population bottleneck in 1989 (Fig. 1B). Subsequently, the mean proportion of adults that were immigrants during the focal period of 1993–2018 was only 0.04 (range 0.00–0.17), and the number of immigrants present did not increase during this period (latent (log) scale regression slope β = 0.004, 95%CI −0.021−0.030). However, since the total adult population size decreased during 1993–2018, the proportion of adults that were immigrants increased slightly (Fig. 1B, regression slope β = 0.0013, 95%CI 0.0005−0.0022).

Mean immigrant genetic group coefficient (*q*_i_) across all 2478 focal individuals hatched during 1993–2018 was 0.56 ± 0.14 SD. Hence, these individuals are on average expected to have inherited approximately half their genome from recent (post‐1975) immigrant ancestors. Mean *q*_i_ was 0.42 ± 0.16 SD in 1993, and increased across cohorts to 0.69 ± 0.07 SD in 2018 (regression slope β = 0.0125, 95%CI 0.012−0.013, Fig. 1C). Yet, *q*_i_ varied substantially among individuals within cohorts (Fig. 1C), reflecting individuals’ differing degrees of recent immigrant ancestry.

### QUANTITATIVE GENETIC PARAMETER ESTIMATES

The genetic groups animal model estimated moderate latent‐scale V_A_ in local juvenile survival (Table 1). There was substantial year (i.e., cohort) variance, but little brood variance (Table 1). Consequently, the posterior mode latent‐scale heritability was 0.11, with a lower 95%CI limit that did not converge to zero (Table 1).

**Table 1:** Summary of posterior distributions of (A) variance components and latent‐scale heritability and (B) fixed effects from the genetic groups animal model for local juvenile survival. Fixed effect estimates are regression slopes, except the sex effect is the contrast of males from females. The regression slope on individual genetic group coefficient is the immigrant genetic group effect g. Statistics are the posterior mean and mode and the 95% highest posterior density credible interval (95%CI) on the latent logit scale. For fixed effects the proportion of posterior samples that were negative is also shown (prop<0). Residual variance was fixed to one by convention

(A) Variance components	Posterior mean [mode]	95%CI [prop<0]
Additive genetic variance	0.36 [0.30]	0.001, 0.72
Brood variance	0.19 [0.01]	0.001, 0.53
Natal year (cohort) variance	1.11 [0.94]	0.49, 1.92
Heritability	0.13 [0.11]	0.02, 0.27
(B) Fixed effects		
Immigrant genetic group coefficient	−2.36 [−2.41]	−4.17, −0.37 [0.990]
Coefficient of inbreeding	−8.92 [−9.50]	−12.58, −5.53 [1.000]
Natal year	−0.06 [−0.05]	−0.14, 0.02 [0.921]
Clutch date	−0.01 [−0.01]	−0.02, −0.01 [1.000]
Sex	0.56 [0.52]	0.31, 0.78 [0.000]

The slope of the regression of juvenile survival on individual *q*_i_ (i.e., the value of g) was negative, and the 95%CI did not span zero (Table 1). Hence, individuals that inherited a greater expected proportion of their genome from recent immigrant ancestors were less likely to locally survive to adulthood. There were negative effects of individual *f*_i_ and clutch date, showing that more inbred and later hatched individuals were less likely to locally survive (Table 1). Males were more likely to locally survive than females (Table 1). The posterior mean regression slope on the natal year was also negative, implying that juvenile survival tended to decrease during 1993–2018 controlling for other effects, but the 95%CI spanned zero (Table 1).

### EVOLUTIONARY CHANGE ACROSS YEARS

**Figure 2:**
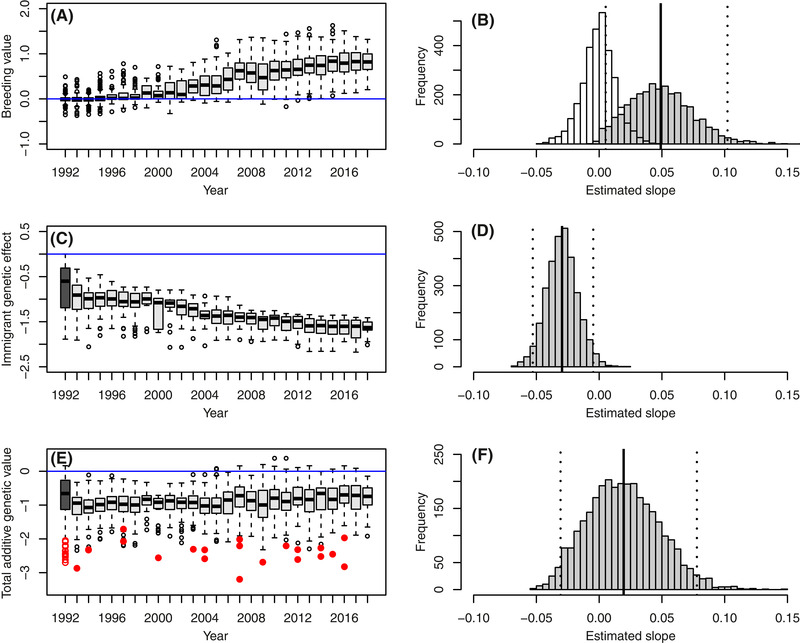
Illustrative summaries of changes in key quantitative genetic parameters across focal cohorts. Mean posterior mode **(A)** breeding values (*a*_i_), **(C)** immigrant genetic effects (g.*q*_i_) and **(E)** total additive genetic values (*u*_i_), and full posterior distributions of regression slopes of **(B)** breeding values (*a*_i_), **(D)** immigrant genetic effects (g.*q*_i_) and **(F)** total additive genetic values (*u*_i_) on year. In (A), (C), and (E), light grey boxplots (specifications as in Figure 1C) summarize estimates for individuals hatched on Mandarte during 1993–2018. Estimates for ancestors hatched before 1993 are pooled into a single category attributed to 1992 for illustration (dark grey). Blue lines denote zero. On E, red points denote immigrants that arrived since 1993 (filled symbols) or previously (open symbols, attributed to 1992 as above). Note that A, C, and E are solely for illustration; temporal changes in posterior modes of individual values were not analysed directly. On B, D, and F, grey histograms show the full posterior distributions of regression slopes, and solid and dotted black lines show posterior means and 95% credible intervals. X‐axis scales are standardized to facilitate comparison. On B, the blue histogram shows the posterior distribution of regression slopes given inheritance of random initial breeding values on the observed pedigree given posterior samples of V_A_. Full posterior distributions are further depicted in Supporting Information S4 and S6.

As expected given nonzero V_A_ in juvenile survival and consistent directional selection, mean breeding value (*a*_i_) for juvenile survival increased during 1993–2018 (illustrated in Fig. 2A). The posterior mean slope of the regression of *a*_i_ on natal year was 0.05 (95%CI: 0.01‐0.10; posterior probability of a positive slope: 0.996; Fig. 2B), and exceeded that arising by chance (i.e., drift) given the observed pedigree (posterior probability of a positive difference: 0.985, Supporting Information S4). The estimated increase in mean *a*_i_ during 1993–2018 was biologically substantial, translating into posterior mean predicted increases in juvenile survival probability of 0.14 (95%CI 0.01‐0.32) and 0.19 (95%CI 0.02‐0.42) during 1993–2018 for females and males given constant mean values of all other modeled variables.

However, since mean immigrant genetic group coefficient (*q*_i_) increased across years (Fig. 1C) and the estimated genetic group effect g was negative (Table 1), the contribution of recent immigrants to individuals’ total additive genetic values (*u*_i_) for local juvenile survival (i.e. g.*q*_i_) became increasingly negative during 1993–2018 (illustrated in Fig. 2C). The posterior mean slope of the regression of g.*q*_i_ on year was −0.03 (95%CI: −0.05 – −0.01; posterior probability of a positive slope: 0.010; Fig. 2D). This decrease was also biologically substantial, with posterior mean decreases in juvenile survival probability due to additive genetic effects of immigrants of 0.06 (95%CI 0.01–0.14) and 0.10 (95%CI 0.01–0.19) for females and males during 1993–2018 given constant mean values of all other modeled variables.

Consequently, since *a*_i_ increased across years and g.*q*_i_ decreased across years, the estimated change in total additive genetic value (*u*_i_) for local juvenile survival was relatively small (illustrated in Fig. 2E). The posterior mean slope of the regression of *u*_i_ on year was 0.02, but the 95%CI spanned zero (−0.03−0.08; posterior probability of a positive slope: 0.738; Fig. 2F). The estimated micro‐evolutionary increase in *a*_i_ across years (Fig. 2A) was therefore substantially counter‐acted by negative additive genetic effects resulting from recent immigration (Fig. 2C), yielding approximate migration‐selection balance (Fig. 2E).

### ADDITIVE GENETIC VARIANCE AND SKEW

**Figure 3:**
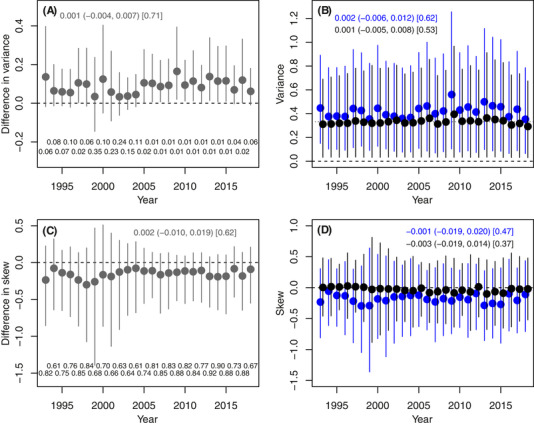
Summaries of posterior distributions of (A) the difference in the variance in *u*_i_ versus the variance in *a*_i_; (B) the variances in *u*_i_ (blue) and *a*_i_ (black); (C) the difference in the skew in *u*_i_ versus the skew in *a*_i_; and (D) the skew in *u*_i_ (blue) and in *a*_i_ (black) across all individuals in each cohort during 1993–2018. Points show posterior means, with 95% credible intervals. Dashed black lines denote zero. In (B), the dotted black line denotes the direct base population estimate of V_A_, adjusted for mean coefficient of inbreeding during 1993–2018 (V_A_(1‐*F*), with *F* = 0.078). In (A) and (C), differences were calculated across pairwise posterior samples. Statistics shown above the plots are the posterior mean change across cohorts, with 95% credible interval (in parentheses) and posterior probability that the regression slope was positive (in square brackets). Statistics below the plots show the posterior probability that each cohort difference is negative.

As expected, the estimated population variance in *u*_i_ across individuals within each cohort exceeded the estimated population variance in *a*_i_ (Fig. 3), representing increases of 10–40%. Immigration therefore increased the total additive genetic variation for juvenile survival that is potentially available to generate a micro‐evolutionary response to selection. Distributions of *u*_i_ also tended to be slightly more negatively skewed than distributions of *a*_i_, but the degree of skew was still small (Fig. 3). The variance and skew in *u*_i_ and *a*_i_, and the differences between them, did not change substantially across the focal cohorts (Fig. 3).

## Discussion

Immigration into any focal population could increase local additive genetic variation and skew, yet could have positive or negative effects on local mean fitness and hence on population growth rate and potential for ‘evolutionary rescue’ (Lenormand 2002; Bell and Gonzalez 2011; Edelaar and Bolnick 2012; Kremer et al. 2012; Gomulkiewicz and Shaw 2013; Carlson *et al*. 2014; McDonald and Yeaman 2018). Scenarios where immigration negates local micro‐evolutionary increases in key adaptive traits and fitness that are otherwise expected given non‐zero V_A_, leading to migration‐selection balance and phenotypic stasis rather than adaptive evolutionary change, are central to evolutionary theory (e.g., Phillips 1996; Yeaman and Guillaume 2009; McDonald and Yeaman 2018). However, dynamic impacts of natural immigration on the mean, variance, and skew in additive genetic values in recipient populations have not been explicitly quantified in wild systems. Leading studies of migration‐selection balance in quantitative traits instead typically focus on understanding observed patterns of phenotypic divergence among (sub)populations (e.g., Postma & van Noordwijk 2005; Yeaman and Jarvis 2006; Moore et al. 2007).

Our quantitative genetic analyses of long‐term song sparrow data show that mean breeding value (i.e., mean *a*_i_) for local juvenile survival increased across years, representing a micro‐evolutionary increase that should be (qualitatively) expected given moderate V_A_ and consistent directional selection. However, this increase was substantially counteracted by introgression from recent immigrants with low mean breeding values (i.e., negative g). Introgression increased the total additive genetic variance (V_U_), but greatly reduced the increase in mean total additive genetic value (i.e., mean *u*_i_) across cohorts that would otherwise have occurred, generating approximate migration‐selection balance. Since V_A_ in juvenile survival accounts for most V_A_ in lifetime reproductive success in song sparrows, particularly in females (Wolak et al. 2018), these results imply approximate migration‐selection balance in fitness. They help explain why there has been no evident major increase in phenotypic juvenile survival (Supporting Information S2), or hence population growth rate and resulting population size (Fig. 1B) across recent years, despite nonzero V_A_ and V_U_. They, therefore, illustrate that raw estimates of additive genetic variation, such as are increasingly available from wild populations, cannot necessarily be straightforwardly used to predict micro‐evolutionary change and population outcomes without further understanding of key processes, such as effects of immigration, that can maintain overall variation yet constrain evolution.

Conceptually, models such as ours that consider individual *q*_i_ as a continuous variable representing an individual's degree of recent immigrant ancestry (Quaas 1988; Westell et al. 1988; Wolak and Reid 2017) extend theoretical quantitative genetic treatments of migration‐selection balance and resulting skew in additive genetic values that categorize individuals as immigrants, natives and F1 offspring (e.g., Yeaman and Guillaume 2009). Yet, previous studies aiming to estimate V_A_ and quantify micro‐evolutionary changes in wild populations have not explicitly used such methods to estimate or account for additive genetic effects of immigrants (or other individuals with unknown parents, Hadfield et al. 2010; Wolak and Reid 2017). This omission has the potential to cause problems with inferring micro‐evolutionary change, and associated attributions of apparent phenotypic stasis to negative environmental counter‐gradients (e.g., Merilä *et al*. 2001). If there is indeed local micro‐evolutionary change, and/or immigrants have mean breeding values that differ from the defined native mean, then standard animal models that do not explicitly account for immigrants can over‐estimate V_A_ (Wolak and Reid 2017). This could cause estimates of micro‐evolutionary change in mean *a*_i_ to be upwardly biased, which will in turn cause inevitable over‐estimation of negative environmental counter‐gradients if mean phenotypes have not in fact changed.

This potential problem is illustrated by repeating our current analyses without fitting an explicit immigrant effect within the animal model (i.e., excluding the regression on *q*_i,_ and hence without estimating g). Posterior mean V_A_ in local juvenile survival is then approximately 47% greater, resulting in greater estimated micro‐evolutionary increases across cohorts and a stronger negative environmental counter‐gradient (Supporting Information S7). These apparently biased inferences, stemming from a model that inappropriately assumes an unstructured base population with the same mean *a*_i_ for native founders and subsequent immigrants, match patterns of microevolution with negative environmental counter‐gradients reported in some other systems (e.g., reviewed in Merilä *et al*. 2001; Pujol et al. 2018). Future quantitative genetic studies aiming to quantify and interpret micro‐evolutionary change should therefore explicitly account for the effects of immigrants (and other forms of base population structure, Wolak and Reid 2017). Our analyses show how this can be achieved, thereby also revealing the degree of migration‐selection balance. Such studies could eventually provide much needed cumulative insights into when immigration is generally likely to increase or decrease the potential for evolutionary change and evolutionary rescue in nature (e.g., Kremer et al. 2012; Gomulkiewicz and Shaw 2013; Bell et al. 2019), at least across micro‐evolutionary timeframes for which pedigree‐based analyses are appropriate (i.e., tens of generations).

### MECHANISMS

Approximate migration‐selection balance arose in song sparrows because recent immigrants had low mean breeding values for local juvenile survival (i.e., g<0). Such negative g could reflect some combination of local adaptation and/or non‐random immigration or heritable dispersal. These mechanisms have been shown or suggested to occur in diverse systems (Lenormand 2002; Postma & van Noordwijk 2005; Doligez and Pärt 2008; Hereford 2009; Edelaar and Bolnick 2012; Savolainen et al. 2013), but cannot be distinguished with our current dataset. Given the substantial effective immigration, local adaptation appears unlikely. Nevertheless, genomic analyses of four sparrow species have shown evidence of genetic divergence at candidate loci for local environmental tolerance, consistent with relatively small‐scale local adaptation (Walsh et al. 2019), and there is some phenotypic evidence of co‐adapted gene complexes on Mandarte (Marr et al. 2002). However, although the quantitative genetic theory of migration‐selection balance typically focuses on local adaptation as the cause of migration load (Yeaman and Guillaume 2009; but see Phillips 1996), various forms of non‐random gene flow could potentially have similar effects without requiring local adaptation (Edelaar and Bolnick 2012).

It is also useful to consider why immigrants’ expected genetic contributions to the focal population are high and increasing further (Fig. 1C), even though immigrants have low mean breeding values for local juvenile survival and hence might be expected to make relatively small long‐term genetic contributions to the local population. All else being equal, the genetic contributions of immigrants to any population relative to a defined historical baseline will inevitably increase over time given continued effective immigration, unless selection against immigrants’ descendants is extremely strong. Nevertheless, mean immigrant contributions could potentially show short‐term dynamics shaped by multiple nonexclusive processes, including changing immigration rate, the local fitness of observed immigrants themselves (which by definition have already survived to adulthood and hence avoided or overcome the hurdle of low local juvenile survival), and any initial heterosis (i.e., non‐additive genetic effects) and resulting relatively high recruitment and/or reproductive success of F1 offspring of immigrant‐native pairings despite their low predicted breeding values. Indeed, previous analyses showed that, while song sparrow immigrants themselves did not have notably high or low reproductive success compared to preexisting natives, there was evidence of heterosis manifested as increased local survival and subsequent reproductive success of recruited F1 offspring (Marr et al. 2002, using data up to 2000). Current estimates of strong inbreeding depression in juvenile survival (Table 1) are also consistent with such effects, since F1 offspring are defined as outbred (see also Reid et al. 2014). In general, heterosis can increase the effective immigration rate, and contribute to initial ‘genetic rescue’ (Ingvarsson and Whitlock 2000; Tallmon et al. 2004). Explicit analyses of the performance of immigrants’ descendants across successive generations are now required to quantitatively partition the dynamics of immigrants’ direct and/or inter‐generational genetic contributions during 1993–2018, including in the context of substantial evident environmental (among‐year) variation and local inbreeding and inbreeding depression (Table 1; Supporting Information S2 and S3), and thereby dissect the demographic basis of the approximate migration‐selection balance. Such comprehensive understanding of the multi‐generational fitness consequences of immigration through both additive and non‐additive genetic effects has rarely been achieved in any wild system, but our analyses highlight that such understanding is required before meaningful quantitative prediction of micro‐evolutionary dynamics in spatially‐structured populations can be achieved.

## AUTHOR CONTRIBUTIONS

J.M.R. conceived and undertook the analyses and drafted the manuscript. P.A. oversaw long‐term data collection. P.N. and L.F.K. undertook pedigree construction, and contributed to fieldwork along with J.M.R. S.M. and M.E.W. contributed to analyses. All authors contributed substantially to conceptual development and manuscript editing.

## DATA ARCHIVING

Data are available from the Dryad digital repository: https://doi.org/10.5061/dryad.rjdfn2z95

Associate Editor: A. Charmantier

## Supporting information


**Appendix S1**. Variance and skew in total additive genetic value.
**Appendix S2**. Summary of local survival probabilities.
**Appendix S3**. Further details of pedigree and covariate data.
**Appendix S4**. Further details of quantitative genetic analyses.
**Appendix S5**. Multi‐collinearity, sampling correlations and data structure.
**Appendix S6**. Phenotypic selection on local juvenile survival.
**Appendix S7**. Models without immigrant genetic effects.
