# Supplementary material for: Immigration counter‐acts local micro‐evolution of a major fitness component: Migration‐selection balance in free‐living song sparrows
Source: Evol Lett. 2021 Jan 15;5(1):48–60. doi: 10.1002/evl3.214 (PMC7857281; doi:10.1002/evl3.214)
Supplement: Supplementary file 1 — Appendix S1. Variance and skew in total additive genetic value. Appendix S2. Summary of local survival probabilities. Appendix S3. Further details of pedigree and covariate data. Appendix S4. Further details of quantitative genetic analyses. Appendix S5. Multi‐collinearity, sampling correlations and data structure. Appendix S6. Phenotypic selection on local juvenile survival. Appendix S7. Models without immigrant genetic effects. [file EVL3-5-48-s001.docx]

**Supporting Information**

**Immigration counter-acts local micro-evolution of a major fitness component: migration-selection balance in free-living song sparrows**

Jane M. Reid, Peter Arcese, Pirmin Nietlisbach, Matthew E. Wolak, Stefanie Muff, Lisa Dickel & Lukas F. Keller

**Appendix S1. Variance and skew in total additive genetic value.**

Given *u*_i_ = *a*_i_ + g*.q*_i_ (main Equation 1) where g is a fixed (i.e. constant) difference between genetic groups) and individual genetic group coefficient *q*_i_ and breeding value *a*_i_ are taken as independent variables, the variance in total additive genetic value V_U_ can be written as a function of the additive genetic variance (V_A_) and the variance in individual *q*_i_ (V_q_) alongside g, where:

V_U_ = V_A_ + g^2^·V_q_ Equation S1 (Reid & Arcese 2020).

Similarly, the skew in total additive genetic value S_U_ can be written as:

S_U_ = S_q_ / (V_A_ + g^2^·V_q_)^3/2^ Equation S2

where S_q_ is the skew in *q*_i_.

These expressions assume that covariance between *q*_i_ and *a*_i_ is zero, which should be approximately true. Expressions for V_U_ and S_U_ that relax this assumption can be derived, and include additional covariance and coskew terms. However, for current purposes, Equations S1 and S2 are presented solely to highlight key components that contribute to V_U_ and S_U_. Values presented in the main text were directly calculated as moments of the full posterior distribution of *u*_i_, thereby directly incorporating any covariance and coskew.

**Appendix S2.** **Summary of local survival probabilities.**

**Figure S1.** Local juvenile survival probabilities for female (blue) and male (black) song sparrow cohorts hatched during 1993-2018 (i.e. local survival from independence from parental care to adulthood the following April). Annual local adult survival probabilities (grey, with females and males pooled) are shown for comparison. Among-year variation is correlated across classes (correlation coefficients: juvenile females and males: 0.75; adults and juvenile females: 0.79; adults and juvenile males: 0.70), reflecting variation in local environmental conditions. Annual local survival probabilities of juveniles are approximately half those of adults (means of 42% and 55% for females and males respectively). These values concur with the rule of thumb that true juvenile survival probabilities are often approximately half adult survival probabilities. Given that adults are apparently highly philopatric, this implies that juvenile emigration is likely to be relatively infrequent with less emigration than philopatry; true juvenile survival probabilities would otherwise be unusually high.


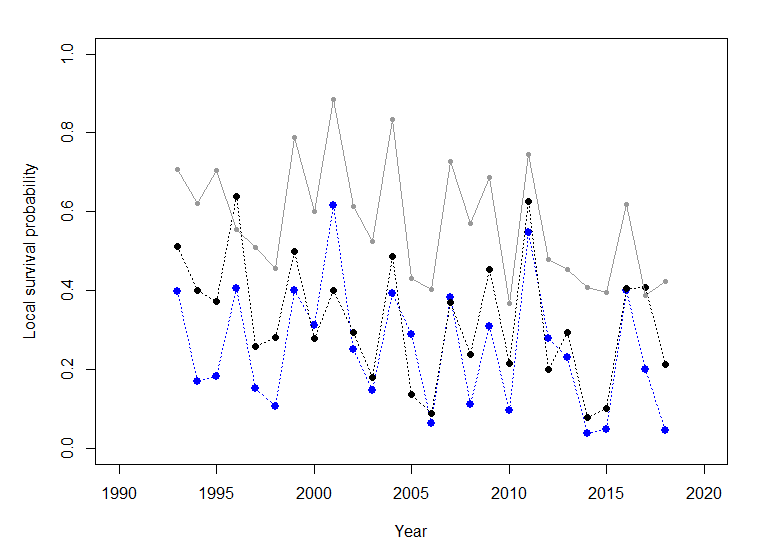


**Appendix S3.** **Further details of pedigree and covariate data.**

**Pedigree data**

Genetic groups animal models and their interpretations fundamentally depend on the defined genetic groups and hence pedigree base populations (Wolak & Reid 2017; Muff et al. 2019). This is because the key relatedness matrix (i.e. matrix of pairwise kinship coefficients) and individual genetic group coefficients (*q*_i_) are estimated relative to the defined base populations (Wolak & Reid 2017).

Further dependencies arise when regressions on individual coefficient of inbreeding (*f*_i_) are also modelled to account for inbreeding depression. Individuals’ *f*_i_ values are estimated relative to the defined native base population, assuming that subsequent immigrants are outbred, unrelated to existing natives at the time of arrival, and unrelated to each other. Consequently, most individuals in initial cohorts have values of *f*_i_=0, and most non-immigrants have values of *q*_i_=0. These values are correct as defined, but may hinder estimation of inbreeding depression (i.e. the slope of the regression on *f*_i_) and the immigrant genetic group effect g (i.e. the slope of the regression on *q*_i_). This is because initial individuals are unlikely to be truly outbred (i.e. have unrelated parents) when native founders are simply taken as the individuals present at an arbitrary study start date in a small population. This is incompatible with the simultaneous assumption that all initial individuals have zero genetic contribution from previous immigrants (i.e. *q*_i_=0). Further, this situation generates positive collinearity between *f*_i_ and *q*_i_, since both are zero for initial individuals and subsequently increase across cohorts. These problems can be resolved by restricting phenotypic data (and hence covariates *f*_i_ and *q*_i_) to individuals with some depth of known ancestry (e.g. all known grandparents is often taken as a minimum, but all known great- or great-great-grandparents may be preferable when a key aim is to distinguish effects of *f*_i_ and *q*_i_).

Accordingly, we restricted phenotypic data to focal individuals hatched on Mandarte since 1993, but utilised pedigree data encompassing all assigned ancestors back to 1975. This provides 17 years (i.e. 6-7 song sparrow generations on average) of pedigree ‘burn-in’. Median *q*_i_ and *f*_i_ for the 1993 cohort are then estimated as approximately 0.4 and 0.05 respectively (Figure 1C, Figure S2), and were weakly negatively correlated across all focal individuals hatched during 1993-2018 (correlation coefficient: -0.2). While the pre-1993 pedigree data presumably contains some paternity error, all mothers and approximately 72% of fathers are likely to be correctly assigned (given zero extra-pair maternity and approximately 28% extra-pair paternity, as observed since 1993). This is a lower error rate than in most wild population pedigrees (e.g. even with partial genetic paternity assignment with 80% population-level confidence). Utilising the pre-1993 pedigree data is therefore preferable to the alternative assumption that all parents of the 1993 cohort were unrelated, and hence all individuals hatched in 1993 had *q*_i_=0 and *f*_i_=0 (e.g. Reid et al. 2011).

Across all focal individuals, mean *f*_i_ was 0.078±0.054SD, and increased only slightly across cohorts (linear regression slope: β=0.0013±0.0001 year^-1^, 95%CI 0.0010–0.0016, Figure S2). Immigration therefore played an important role in maintaining local population fitness by preventing the otherwise inevitable increase in local inbreeding, but such effects are not the primary focus of current analyses. Table S1 summarises the cohort distributions of *f*_i_ and *q*_i_. Figure S3 shows the distribution of pairwise coefficients of kinship between all phenotyped individuals (mean 0.071±0.035SD). This relatively high mean and substantial variance, and underlying high connectedness of the pedigree across years, provide substantial power for quantitative genetic analyses. As with all pedigree-based quantitative genetic analyses, values of *q*_i_, *f*_i_ and pairwise kinship are expectations given the observed pedigree and do not capture deviations due to Mendelian sampling (i.e. drift) or selection. However, such deviations are generally expected to be relatively small for highly polygenic traits (i.e. assuming weak selection on alleles at any locus).

**Figure S2.** Distributions of individual coefficient of inbreeding (*f*_i_) and clutch dates across focal cohorts hatched in 1993-2018. Boxplots show the median (central thick line), first and third quantiles (box limits), 1.5x interquartile range (whiskers) and outliers (points).


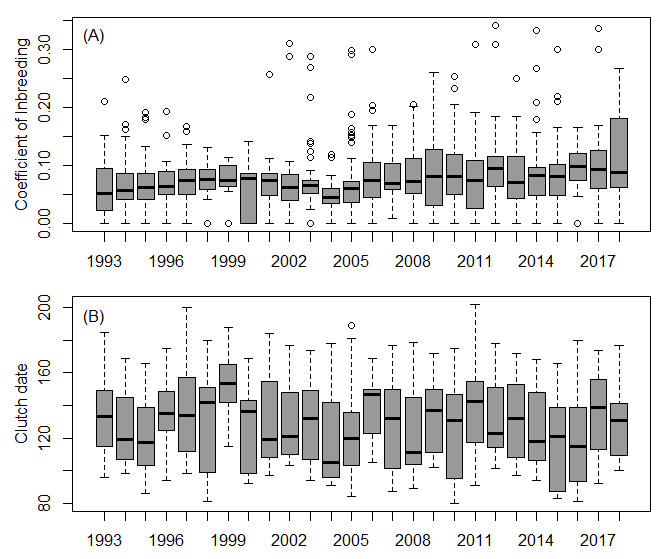


**Table S1.** Cohort-specific mean, variance and skew in individual genetic group coefficient (*q*_i_) and coefficient of inbreeding (*f*_i_).

|  | **Genetic group coefficient (*q*_i_)** | | | **Coefficient of inbreeding (*f*_i_)** | | |
| --- | --- | --- | --- | --- | --- | --- |
| **Cohort** | **Mean** | **Variance** | **Skew** | **Mean** | **Variance** | **Skew** |
| 1993 | 0.416 | 0.024 | 0.573 | 0.062 | 0.002 | 0.599 |
| 1994 | 0.425 | 0.012 | 0.625 | 0.064 | 0.002 | 1.911 |
| 1995 | 0.422 | 0.011 | 0.625 | 0.066 | 0.001 | 1.082 |
| 1996 | 0.417 | 0.010 | 0.764 | 0.068 | 0.001 | 1.166 |
| 1997 | 0.445 | 0.012 | 0.993 | 0.073 | 0.001 | 0.413 |
| 1998 | 0.461 | 0.015 | 1.103 | 0.072 | 0.001 | -0.700 |
| 1999 | 0.440 | 0.014 | 1.371 | 0.077 | 0.001 | -0.963 |
| 2000 | 0.509 | 0.020 | 0.586 | 0.061 | 0.002 | -0.288 |
| 2001 | 0.492 | 0.014 | 1.219 | 0.080 | 0.004 | 1.698 |
| 2002 | 0.497 | 0.009 | 0.950 | 0.071 | 0.004 | 2.605 |
| 2003 | 0.513 | 0.007 | 0.868 | 0.072 | 0.002 | 2.618 |
| 2004 | 0.564 | 0.007 | 0.819 | 0.049 | 0.001 | 1.030 |
| 2005 | 0.591 | 0.011 | 0.573 | 0.068 | 0.004 | 1.805 |
| 2006 | 0.588 | 0.007 | 0.425 | 0.079 | 0.003 | 1.041 |
| 2007 | 0.589 | 0.005 | 1.056 | 0.076 | 0.001 | 0.314 |
| 2008 | 0.603 | 0.005 | 1.015 | 0.089 | 0.003 | 0.694 |
| 2009 | 0.643 | 0.008 | 0.672 | 0.081 | 0.004 | 0.753 |
| 2010 | 0.618 | 0.006 | 0.993 | 0.087 | 0.003 | 0.518 |
| 2011 | 0.647 | 0.006 | 0.660 | 0.077 | 0.004 | 1.454 |
| 2012 | 0.636 | 0.005 | 0.989 | 0.097 | 0.005 | 1.734 |
| 2013 | 0.670 | 0.008 | 0.750 | 0.080 | 0.003 | 0.461 |
| 2014 | 0.674 | 0.007 | 0.733 | 0.083 | 0.005 | 1.834 |
| 2015 | 0.678 | 0.007 | 0.764 | 0.079 | 0.003 | 1.126 |
| 2016 | 0.676 | 0.005 | 0.226 | 0.101 | 0.002 | -0.334 |
| 2017 | 0.689 | 0.009 | 0.610 | 0.092 | 0.005 | 1.085 |
| 2018 | 0.685 | 0.005 | 0.796 | 0.113 | 0.006 | 0.515 |

**Figure S3.** Distribution of pairwise coefficients of kinship between all 2478 phenotyped individuals. Mean 0.071, median 0.064, standard deviation 0.035, 95% range 0.003-0.457 across 6138006 non-self pairwise comparisons (i.e. 2478^2^-2478).


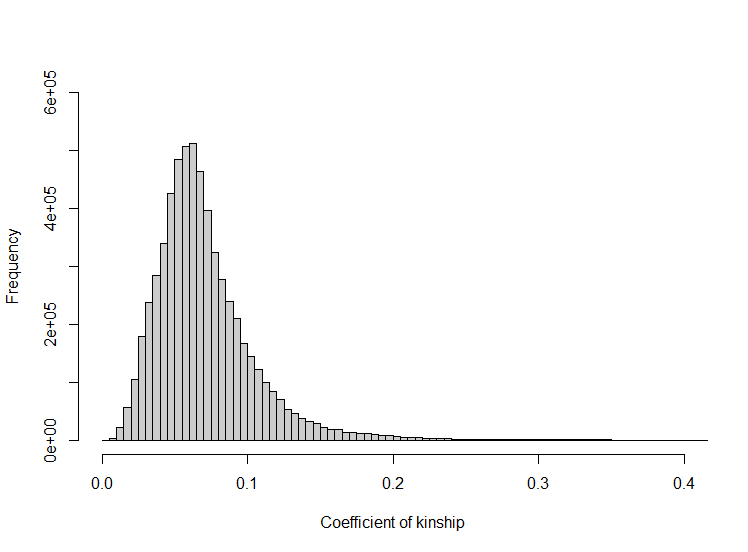


**Clutch date**

Clutch date for each focal individual (i.e. lay date of the first egg in the focal individual’s natal clutch) was directly observed for nests found during building and laying, or back-calculated from hatch dates and/or chick biometrics for nests found subsequently. Cohort-specific distributions are shown in Figure S2. Note that since song sparrows commonly produce multiple clutches per season, clutch date often does not equal the first seasonal laying date of the parents (which is commonly the focus of studies of reproductive phenology, e.g. Germain et al. 2016). Clutch date did not change markedly across years, but if anything tended to get slightly earlier (linear regression slope: β = -0.15±0.10SE days year^-1^, 95%CI -0.34–0.04).

**Sex assignments**

Sexes of all focal individuals that survived to adulthood were accurately assigned based on reproductive behaviour (male song, female incubation). Sexes of most individuals that did not survive to adulthood were assigned by genotyping the CHD1 locus (Postma et al. 2011). Only 6 individuals hatched during 1993-2016 have not been CHD1-genotyped, and for current analyses these were randomly assigned as female or male. However 110 individuals hatched in 2017-2018 (comprising most individuals from these cohorts) have not yet been CHD1-genotyped. These individuals were randomly assigned as female using a Bernoulli trial with probability *s*_f_, where *s*_f_ = (*p*_p_-(1-*p*_u_).*p*_f_)/*p*_u_, or male otherwise. Here, *p*_p_ is the true probability that a hatchling would be female, and was taken to be 0.5 given the previously observed 1:1 primary sex ratio (Postma et al. 2011). *p*_f_ is the proportion of individuals from each cohort that survived to adulthood (and hence whose sex was directly observed) that were female. *p*_u_ is the proportion of each cohort that did not locally survive to adulthood and hence was of unknown sex. This formulation retained the correct (known) sex-specific local survival probabilities for each cohort, thereby allowing correct estimation of the fixed effect of sex (i.e. the mean difference in local juvenile survival probability of females and males). Since sex-specific additive genetic variances and sex-specific effects of immigrants were not estimated in current analyses (see Wolak et al. 2018 for such analyses), such random sex assignment for dead individuals could not cause error or bias in current analyses.

**Appendix S4.** **Further details of quantitative genetic analyses.**

**Genetic groups animal model**

Application of pedigree-based mixed model analyses (i.e. ‘animal models’) has revolutionised quantitative genetic analyses of wild population data, not least because such models can incorporate effects of selection (Kruuk 2004), inbreeding (Reid & Keller 2010) and immigration or other forms of base population structure (Wolak & Reid 2017).

For current analyses, the genetic groups animal model was of the general form:

**l** = **Xβ** + **Za** + **Zy_r_** + **Zb**

where l is a vector of latent variable values underlying juvenile survival; a, y_r_ and b are vectors of additive genetic, year (cohort) and natal brood random effects; β is a vector of specified fixed effects; and X and Z are the respective design matrices. Random parental effects were not fitted because previous analyses showed that associated parental environmental variances in juvenile survival are small (Reid 2012; Wolak et al. 2018). Residual (i.e. overdispersion) variance is not identifiable for binary traits, and was fixed to one by convention.

The fixed effects structure was specified as:

**β_g_.*q* + β_f_.*f* + β_d_.d + β_y_.y_c_ + β_s_.s**

where: β_g_ is the regression slope on immigrant genetic group coefficient (*q*) across individuals, and equals the immigrant genetic group effect g.

β_f_ is the regression slope on individual coefficient of inbreeding (*f*) across individuals, and captures inbreeding depression (and potentially also effects of heterosis).

β_d_ is the regression slope on clutch date (d) across individuals.

β_y_ is the regression slope on natal year (specified as a covariate, y_c_).

β_s_ is a fixed effect of sex (specified as a factor, s).

Previous analyses indicated no strong genetic group by sex interaction, so such effects were not considered further (Wolak et al. 2018). Conclusions remained similar when the regression on natal year was removed, and when analyses were repeated using the unpruned pedigree. Estimates of additive genetic variance and the genetic group effect g, which are of current primary interest, also remained similar when the models included an additional fixed effect that described whether or not a focal individual had an immigrant parent.

**Model code for R package MCMCglmm**

prior = list(R = list(V = 1, fix = 1), G = list(G1 = list(V = 1, nu = 1, alpha.mu = 0, alpha.V = 1000), G2 = list(V = 1, nu = 1, alpha.mu = 0, alpha.V = 1000), G3 = list(V = 1, nu = 1, alpha.mu = 0, alpha.V = 1000)))

model.gg <- MCMCglmm(surv.ind.to.ad ~ f.coef + q.coef + natalyr.cont + clutch.date + sex.factor,

random = ~ animal + brood.id + natalyr.factor, family = "categorical",

data = data, prior = prior, pedigree = pruned.pedigree, verbose = TRUE,

pr = TRUE,

burnin = 5000, nitt = 3005000, thin=1000)

**Key:**

Dependent variable:

surv.ind.to.ad: Binary variable describing whether each focal individual survived from independence from parental care to adulthood the following April. Immigrants’ own phenotypes for juvenile survival were excluded, since any immigrants that did not survive were not observed.

Fixed effects:

f.coef: Vector of individual coefficient of inbreeding (*f*_i_), pre-calculated from the pedigree.

q.coef: Vector of individual immigrant genetic group coefficient (*q*_i_), pre-calculated from the pedigree.

natalyr.cont: Each individual’s natal year, specified as a continuous variable (with 1975 coded as year 1).

clutch.date: Lay date of each individual’s natal clutch, specified as a continuous variable.

sex.factor: Binary variable specifying whether an individual was assigned as female or male.

Random effects:

animal: Individual identity, linked to variance-covariance structure defined by the inverse relatedness matrix computed from the pedigree.

brood.id: Individual’s natal brood identity.

natalyr.factor: Individual’s natal year specified as a factor.

pr = TRUE saves posterior samples of random effects, including breeding values for all individuals included in the pedigree.

**Alternative genetic group formulations**

Results and conclusions remained similar given a more time-restricted definition of the immigrant genetic group, where five individuals that arrived before 1988 were classed as natives. This is because these immigrants made small genetic contributions to cohorts hatched since 1993, with little variation among focal individuals. The model structure could in principle be extended to include more genetic groups, for example to consider different groups of immigrants blocked by time periods. However, fitting too many groups may cause collinearities and hence impede estimation, and exploratory analyses with the current dataset did not show any major temporal structure in immigrants’ values of *a*_i_, or hence *u*_i_, across the focal years. Since any temporal change in additive genetic values of newly-arrived immigrants across years was not explicitly modelled, the estimated overall change in *a*_i_ across cohorts (Figure 2A) may be very slightly conservative (i.e. underestimated).

Standard genetic groups animal models assume equal V_A_ in all specified base populations (here natives and immigrants, Wolak & Reid 2017). These models can be extended to estimate group-specific V_A_, by formulating group-specific relatedness matrices (Muff *et al*. 2019). For the current dataset, such analyses returned very similar posterior mean V_A_ for the native and immigrant groups, albeit with considerable uncertainty. This implies that the standard assumption of equal V_A_ is broadly appropriate. We therefore present results from the standard model, thereby increasing precision and simplifying inference of changing distributions of *a*_i_.

**Simulations of drift**

Evidence that a change in mean breeding value (*a*_i_) across cohorts exceeds zero provides evidence of an evolutionary change, but does not directly prove that the change exceeds that which could have arisen due to drift and hence can be interpreted as a response to selection (Hadfield et al. 2010). To test whether the estimated temporal change in mean *a*_i_ across song sparrow cohorts exceeded that which could arise simply due to Mendelian sampling and inevitable positive autocorrelation across generations (i.e. drift) given the observed pedigree, values of *a*_i_ were simulated on the pedigree using function *rbv* in R package MCMCglmm (Hadfield et al. 2010). Here, *a*_i_ values for all founder individuals in both defined genetic groups are drawn from a normal distribution with mean zero and variance equal to each posterior sample of V_A_, then propagated down the pedigree. Offspring inherit the mean of their parents’ values plus a deviation drawn from a normal distribution with mean zero and variance ½V_A_(1-F) (i.e. the assumed Mendelian sampling variance, where F is the mean parental coefficient of inbreeding). These simulations preserve the full observed pedigree structure, including the natural pattern of immigration and subsequent reproductive success of all individuals. Changes in mean simulated (i.e. random) *a*_i_ across years are therefore directly and quantitatively comparable to the change in mean *a*_i_ estimated from the real data.

However, when the focal trait is closely linked to fitness (as is juvenile survival), the observed pedigree is itself shaped by selection and should contain more variance in lineage size than expected if variation in survival were solely due to environmental and demographic stochasticity. We therefore additionally repeated the above simulations but, within each iteration, restructured the pedigree by randomly reassigning (i.e. resampling without replacement) observed parents to offspring within each cohort. These simulations randomised lineage structure while retaining observed distributions of reproductive success and immigration, and hence did not alter the mean degree of inbreeding.

The regression slope of observed *a*_i_ on natal year exceeded those arising by chance both given the observed pedigree, and given the randomised pedigrees (posterior probabilities of positive differences: 0.985 and 0.990 respectively, Figure S4). These distributions of the difference in slope between observed and simulated changes in *a*_i_ include high frequencies of small values (Figure S4). Since the estimated evolutionary change in mean *a*_i_ is quite substantial, this illustrates that there is substantial scope for mean *a*_i_ to increase across generations solely due to drift (as is inevitable given the small size of the focal population).

These simulations of changes in mean *a*_i_ across cohorts assume that V_A_, and hence the Mendelian sampling variance, remain approximately constant across generations. This is likely to be approximately true (assuming that juvenile survival is indeed highly polygenic, Figure 3). However, V_A_ may decrease slightly between the base population and cohorts observed during 1993-2018 due to initial accumulation of inbreeding (mean *f*_i_ is assumed to be zero in the base populations, but was 0.078 during 1993-2018, Figure S2). However, all conclusions remained the same when simulations were repeated taking V_A_(1-*F*) rather than V_A_, with *F* = 0.078. This degree of inbreeding had a scarcely perceptible effect on the estimated magnitude of drift.

**Figure S4.** Posterior distributions of the difference between regression slopes of estimated versus randomised breeding values (*a*_i_) on hatch year given (A) randomised breeding values on the observed pedigree and (B) randomised breeding values on randomised pedigrees. Blue lines demarcate zero. Differences are calculated as paired differences given each posterior sample of additive genetic variance (V_A_) in local juvenile survival.


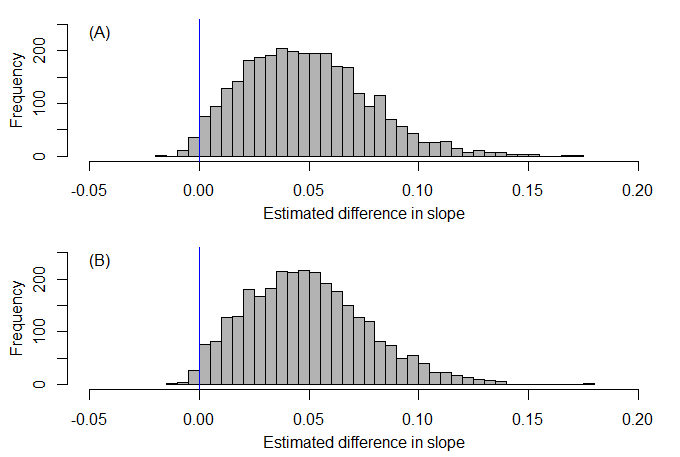


Similarly, evidence that a change in mean total additive genetic value (*u*_i_) across cohorts exceeds zero would provide evidence of overall evolutionary change, but would not directly distinguish a response to selection from drift. A full null (i.e. neutral) model for change in mean *u*_i_ across years is hard to define, because changes in *u*_i_ depend on changes in mean *q*_i_ and hence on the pattern of effective gene flow. But, there is no obvious null (neutral) model for effective gene flow, which will depend on immigration rate, and reproductive success of immigrants in relation to stochastic environmental variation in recruitment. Further, observed changes mean *q*_i_ are a fixed property of the observed pedigree, and hence cannot be altered while retaining the observed pedigree structure and associated degree of inbreeding. However, in current analyses, the change in mean *u*_i_ across years did not differ from zero, providing no evidence of an evolutionary change (either due to drift or selection). We therefore did not currently pursue explicit tests of whether observed changes differed from some neutral expectation. Since *u*_i_ is an additive function of *a*_i_ and the above simulations show that the change in *a*_i_ exceeded that expected due to drift given the observed pedigree, this implies that the (lack of) change in mean *u*_i_ across years is not simply neutral, but represents adaptation counter-acted by recent immigration (i.e. approximate migration-selection balance).

Note that the negative mean *u*_i_ (intercept on main Figure 2E, calculated relative to the mean of zero for the defined native base population) does not necessarily imply that population-wide mean *u*_i_ was lower during 1993-2018 than previously. This is because the observed base population (comprising assigned ancestors of individuals observed during 1993-2018) is likely to comprise individuals with relatively high values of *a*_i_ and *u*_i_, since lineages with low values are more likely to have gone locally extinct during 1975-1992. This is the same process as causes the observed micro-evolutionary increase in *a*_i_ during 1993-2018 (Figure 2A). The pattern of immigration and effective gene flow observed during 1993-2018 might or might not be representative of previous or future years, meaning that longer-term predictions regarding the degree of migration-selection balance cannot be readily made.

**Figure S5.** Summaries of posterior mean (A) breeding values (*a*_i_), (B) immigrant genetic effects (g.*q*_i_) and (C) total additive genetic values (*u*_i_) for each focal cohort. Boxplots show the median (central thick line), first and third quantiles (box limits) and 1.5x interquartile range (whiskers), and blue lines denote zero. This provides an alternative representation of the patterns shown in main figure 2, panels A, C and E.


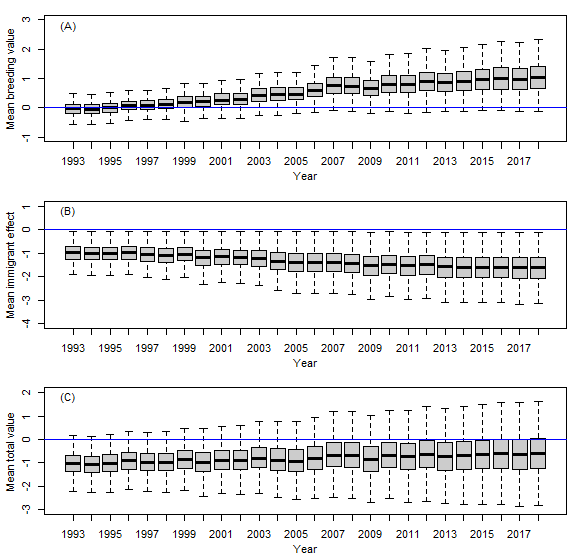


**Appendix S5. Multi-collinearity, sampling correlations and data structure.**

Interpretations of genetic groups animal models that include fixed regressions on genetic group coefficients (*q*_i_) rely on unbiased and relatively precise estimation of regression slopes as well as variance components. As with any multiple regression, estimates can be biased and imprecise if there is multi-collinearity among independent variables. In general, such multi-collinearity may not be a serious problem when the primary aim is prediction as opposed to hypothesis testing, and when uncertainty can be appropriately propagated (as in current analyses). Nevertheless, it is important for current interpretations that effects of the covariates *q*_i_, *f*_i_, clutch date and natal year are not substantially confounded.

Variance inflation factors (VIF), which quantify the degree of inflation in variances of parameter estimates due to collinearities, showed no evidence of major problems due to multi-collinearity (Table S2). Clutch date and sex were entirely independent of the other variables, and *f*_i_ was largely so (VIF values of 1.0 indicate complete independence). Natal year and *q*_i_ were weakly associated, but the VIF value of 2.7 was still relatively low. As rules of thumb, values >4.0 are often deemed sufficiently high to warrant further investigation, while values >10.0 indicate serious multi-collinearity that is likely to substantially affect model estimates.

Nevertheless, to further verify the negative effect of *q*_i_ estimated by the genetic groups animal model fitted to the whole dataset (i.e. g, Table 1), we fitted a series of separate linear regressions to test whether the relationship between *q*_i_ and juvenile survival was also negative within each individual cohort. This approach eliminates any possible confounding caused by any correlated but independent changes in *q*_i_ and juvenile survival across years. These regression analyses also estimated negative effects of *q*_i_ on juvenile survival within most cohorts, implying that individuals within each cohort with high *q*_i_ values are less likely to survive (negative slopes, Figure S6). The exceptions (i.e. positive slopes) typically refer to years with low cohort-wide survival rate (left side of Figure S6), and/or with smaller cohort sample sizes (smaller point sizes). Further, the grand mean regression slope estimated across all individual cohorts almost exactly matched the overall slope (g) estimated by the genetic groups animal model (Figure S6). This implies that the animal model estimate of g, and hence the inferred mean additive genetic effect of immigrants, is not biased by covariance between individual *q*_i_ and natal year. Such negative slopes can be interpreted as evidence of selection against alleles imported by immigrants.

**Table S2.** Variance inflation factors for five covariates (fixed effects) included in the genetic groups animal model.

| **Fixed effect** | **Variance inflation factor** |
| --- | --- |
| Natal year | 2.7 |
| Individual immigrant genetic group coefficient (*q*_i_) | 2.7 |
| Individual coefficient of inbreeding (*f*_i_) | 1.4 |
| Clutch date | 1.0 |
| Sex | 1.0 |

**Figure S6.** Summary of regressions of local juvenile survival from independence to adulthood on individual immigrant genetic group coefficient (*q*_i_) within each cohort. Each point shows the partial regression slope (controlling for individual coefficient of inbreeding, clutch date and sex) for each cohort (y-axis) plotted against the overall survival rate for the focal cohort (x-axis). Point sizes represent the number of focal individuals in each cohort (i.e. the sample size). The colour scale from dark grey to light grey denotes cohorts from 1993-2018. The solid black line denotes zero. The dashed grey line denotes the grand mean across all cohort-specific estimates. For comparison, the dashed black line denotes the genetic group effect (g) estimated by the full genetic groups animal model (main Table 1).


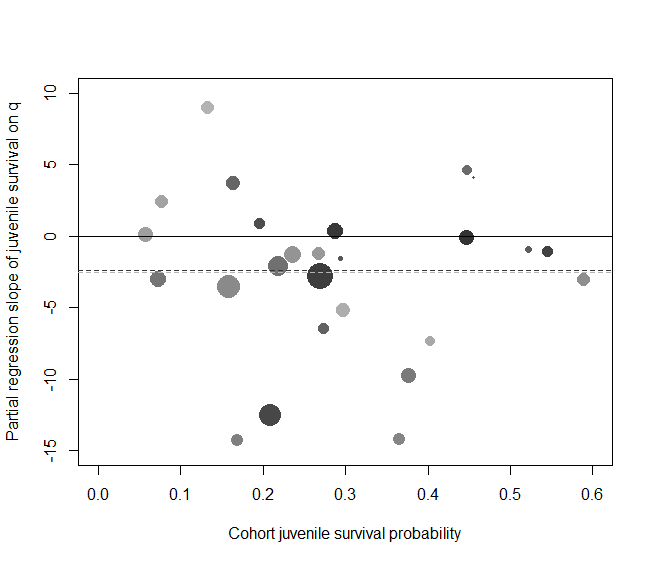


Since individuals inherit both their breeding value *a*_i_ and their genetic group coefficient *q*_i_ from their parents (Wolak & Reid 2017), animal model estimates of g and *a*_i_ (and hence V_A_) could conceivably be partially confounded. However, there is no evidence that this is the case across the current dataset. Individual *q*_i_ was uncorrelated with pairwise kinship across all individuals (correlation coefficient: -0.04), and the magnitude of the pairwise difference in *q*_i_ was only weakly correlated with pairwise kinship (correlation coefficient: 0.22). This is partly because an individual’s *q*_i_ reflects its expected genetic contribution from the defined immigrant group, not from any individual immigrant lineage. Two individuals could consequently have similarly high values of *q*_i_ but be unrelated, for example if they have different immigrant parents. The posterior mean correlation between individual *q*_i_ and *a*_i_ across all focal individuals was positive, as is inevitable since both increased on average across cohorts (0.29; 95%CI 0.02-0.47). However, correlations within cohorts were weak (grand posterior mean: -0.14). The MCMC sampling correlation between V_A_ and g was also small (0.13), as were the sampling correlations between individual *a*_i_ and g (mean 0.14, 95%CI -0.01 – 0.23). Finally, previous analyses of other components of song sparrow fitness estimated values of g close to zero despite non-zero V_A_ (Wolak et al. 2018), proving that the song sparrow data structure does not force g to be negative.

**Appendix S6. Phenotypic selection on local juvenile survival.**

Since individuals that do not survive to adulthood cannot produce any offspring, local phenotypic selection on local juvenile survival must be consistently directional and positive, and should therefore be expected to drive an evolutionary increase in mean breeding value (*a*_i_) for juvenile survival. To indicate the magnitude of selection, we calculated phenotypic selection gradients by regressing individual lifetime reproductive success (LRS) on individual local juvenile survival within each focal cohort. Each individual’s LRS was calculated as the total number of ringed (i.e. ca. 6 day old) offspring it produced on Mandarte over its lifetime. Unstandardised selection gradients simply equal the mean LRS of individuals that survived to adulthood (since mean LRS of individuals that did not survive is always exactly zero). However, to facilitate some comparison across cohorts given that local juvenile survival probabilities varied among years (Figure S1) we also calculated standardised selection gradients with relative LRS (i.e. individual LRS divided by cohort mean LRS) and standardised survival (i.e. individual survival minus cohort mean survival divided by the standard deviation). Females and males were pooled for simplicity. Analyses were restricted to cohorts hatched during 1993-2015, which were completely or almost extinct by 2019 (meaning that all individuals’ LRS could be fully evaluated). Conclusions were similar when analyses were repeated using an individual’s contribution to the population in the year after hatch instead of LRS as the measure of fitness (i.e. S + ½B, where S is individual survival coded 1 or 0 and B is the individual’s reproductive success in its first year).

Figure S7 depicts the standardised and unstandardised selection gradients using LRS for each cohort. Grand mean standardised and unstandardised selection gradients across all cohorts were 1.8 and 9.8 respectively (ranges 0.8 – 4.1 and 4.7 – 17.1 respectively).

**Figure S7.** Illustrations of phenotypic selection gradients on local juvenile survival. Boxplots show cohort-specific distributions of lifetime reproductive success (LRS) of individuals that locally survived to adulthood (median (central thick line), first and third quantiles (box limits), 1.5x interquartile range (whiskers) and outliers (open points)). Filled black points show mean LRS, which equals the unstandardised selection gradient. Blue points show the standardised selection gradient (on the same y-axis scale). The numbers of focal individuals within each cohort that did (bold) and did not locally survive to adulthood are shown. The dotted line denotes zero.


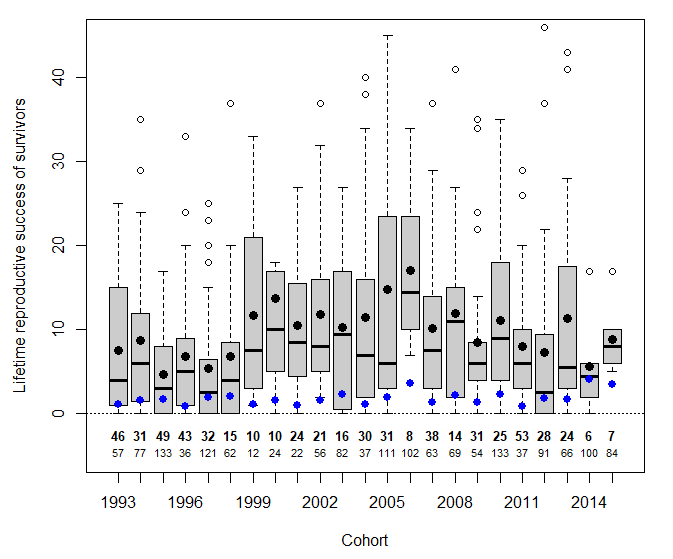


**Appendix S7. Models without immigrant genetic effects.**

We fitted an identical animal model to the same dataset as reported in the main text, except that the immigrant genetic group effect was not included (i.e. no fixed regression on *q*_i_, or hence estimate of g). The posterior mean V_A_ was then ~47% higher than that estimated by the full model including the immigrant effect (Table S3 versus Table 1, although the 95%CI included the posterior mean estimate from the full model).

Mean breeding value (*a*_i_) for juvenile survival was consequently estimated to increase substantially across natal years (illustrated in Figure S8). The posterior mean slope of the regression of *a*_i_ on natal year was 0.06 (95%CI 0.02-0.12; posterior probability of a positive slope: 0.999), and exceeded that which arose by chance given the observed pedigree (posterior probability of a positive difference: 0.997). This degree of evolutionary change exceeded that estimated by the full model that included the immigrant genetic group effect (Figure S8). Consequently, the negative environmental counter-gradient was estimated to be almost twice as strong, with a 95%CI that no longer included zero (fixed effect of natal year in Table S3 versus Table 1).

Note that such upward bias in estimates of V_A_ can readily occur when non-founder individuals with unknown parents (in this case immigrants) are inappropriately assigned to a single unstructured base population (Wolak & Reid 2017). It does not imply that estimates of V_A_ and the immigrant genetic group effect g are directly confounded (see Supporting Information S5).

**Table S3.** Summary of (A) variance components and heritability and (B) fixed effects estimated by an animal model for local juvenile survival that did not include a regression on immigrant genetic group coefficient, or hence explicitly estimate an immigrant genetic group effect. Terms are as in main Table 1. Statistics are the posterior mean and mode and the 95% highest posterior density credible interval (95%CI). For fixed effects the proportion of posterior samples that were negative is also shown (prop<0). All estimates are on the latent logit scale.

| (A) Variance components | Posterior mean [mode] | 95%CI [prop<0] |
| --- | --- | --- |
| Additive genetic variance | 0.53 [0.33] | 0.15, 1.01 |
| Brood variance | 0.18 [0.002] | <0.001, 0.51 |
| Natal year (cohort) variance | 1.13 [0.84] | 0.47, 1.93 |
| Heritability | 0.18 [0.13] | 0.07, 0.32 |
|  |  |  |
| (B) Fixed effects |  |  |
| Immigrant genetic group coefficient | --------------- | ------------------ |
| Coefficient of inbreeding | -7.57 [-7.41] | -10.93, -3.89 [1.000] |
| Natal year | -0.10 [-0.05] | -0.18, -0.03 [0.997] |
| Clutch date | -0.01 [-0.01] | -0.02, -0.01 [1.000] |
| Sex | 0.56 [0.53] | 0.30, 0.79 [0.000] |

**Figure S8.** Illustrative summaries of changes in posterior mode breeding values (*a*_i_) with natal year estimated from animal models that (A) did and (B) did not explicitly estimate the immigrant genetic group effect. Light grey boxplots denote individuals hatched on Mandarte in each year during 1993-2018. These individuals’ ancestors that hatched before 1993 are pooled into a single category attributed to 1992 for simple illustration (dark grey). Boxplot specifications are as in Figure 1C. Y-axis scales are standardised to facilitate comparison. Blue lines denote zero (solid line) or other unit values (dashed lines) to facilitate visual comparison. Note that these figures are solely for simple illustration; temporal changes in posterior modes of individual values were not analysed directly. Panel A is the same data as in figure 2A.


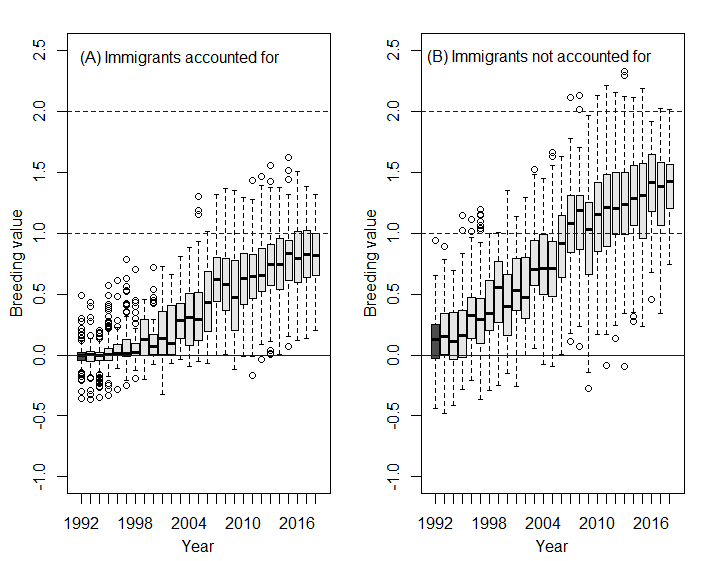


**References**

Germain, R.R., Wolak, M.E., Arcese, P., Losdat, S. & Reid, J.M. (2016). Direct and indirect genetic and fine-scale location effects on breeding date in song sparrows. *J. Anim. Ecol.* 85:1613-1624.

Hadfield, J.D., Wilson, A.J., Garant, D., Sheldon, B.C. & Kruuk, L.E.B. (2010). The misuse of BLUP in ecology and evolution. *Am. Nat*. 175:116-125.

Kruuk, L.E.B. (2004). Estimating genetic parameters in natural populations using the ‘animal model’. *Phil. Trans. R. Soc. B* 359:873-890.

Muff, S., Niskanen, A.K., Saatoglu, D., Keller, L.F. & Jensen, H. (2019). Animal models with group-specific additive genetic variances: extending genetic group models. *Genet. Sel. Evol.* 51:7.

Postma, E., Heinrich, F., Koller, U., Sardell, R.J., Reid, J.M., Arcese, P. & Keller, L.F. (2011). Disentangling the effect of genes, the environment and chance on sex ratio variation in a wild bird population. *Proc. R. Soc. B* 278:2996-3002.

Reid, J.M., Arcese, P., Sardell, R.J. & Keller, L.F. (2011). Additive genetic variance, heritability and inbreeding depression in male extra-pair reproductive success. *Am. Nat.* 177:177-187.

Reid, J.M. (2012). Predicting evolutionary responses to selection on polyandry in the wild: additive genetic covariances with female extra-pair reproduction. *Proc. R. Soc. B* 279:4652-4660.

Reid, J.M. & Keller, L.F. (2010). Correlated inbreeding among relatives: occurrence, magnitude and implications. *Evolution* 64:973-985.

Wolak, M.E. & Reid, J.M. (2017). Accounting for genetic differences among unknown parents in microevolutionary studies: How to include genetic groups in quantitative genetic animal models. *J. Anim. Ecol.* 86:7-20.

Wolak, M.E., Arcese, P., Keller, L.F., Nietlisbach, P. & Reid, J.M. (2018). Sex-specific additive genetic variances and correlations for fitness in a song sparrow (*Melospiza melodia*) population subject to natural immigration and inbreeding. *Evolution* 72:2057-2075.
